# The Critical Role of Trace Elements in Bone Health

**DOI:** 10.3390/nu16223867

**Published:** 2024-11-13

**Authors:** Miaoqian Li, Feidan Deng, Lichun Qiao, Xinyue Wen, Jing Han

**Affiliations:** 1 School of Public Health, Xi’an Jiaotong University Health Science Center, Xi’an 710061, China; l1058427391@163.com (M.L.); dfdmnz@163.com (F.D.); qlc978402409@163.com (L.Q.); 15736116663@163.com (X.W.); 2 Key Laboratory for Disease Prevention and Control and Health Promotion of Shaanxi Province, Xi’an 710061, China

Trace elements are essential for human physiology and crucial in maintaining bone health and regulating bone metabolism. The importance of trace elements in maintaining bone health has garnered escalating attention. These elements play a crucial role in regulating bone mineral density (BMD), preventing osteoporosis, and reducing fracture risk, while also participating in various biological processes that modulate bone metabolism. Research indicates that trace elements impact bone health by influencing cellular activity and regulating bone remodeling, which continually replaces old bone with new. Additionally, trace elements interact with metabolic pathways, modulating the inflammatory response and impacting bone health. Therefore, ensuring sufficient intake and balance of these essential elements is crucial for preventing bone diseases and promoting skeletal health.

This Special Issue of *Nutrients*, entitled “*Trace Elements and Bone Health*”, provides detailed insights into how these trace elements impact bone health, enhancing our understanding of their potential role in preventing and managing bone-related diseases. The Special Issue included six studies: one systematic review, one brief report, and four research studies. Collectively, these delved into the intricate relationship between trace elements and their impact on bone health.

As a significant area of bone research, dentistry has also attracted considerable attention. A systematic review by Dr. Buzatu et al. evaluates the impact of vitamin D on the osseointegration of dental implants, encompassing seven studies conducted between 2008 and 2021, involving 1462 participants and 4450 implants [1]. The findings reveal a strong correlation between low serum vitamin D levels and an elevated risk of early implant failure, particularly among patients who smoke or suffer from periodontal disease. Additionally, the authors suggest that vitamin D supplementation has been shown to improve postoperative bone density, thereby contributing to improved implant stability and success rates. This study underscores the significance of optimizing vitamin D levels to promote oral health and mitigate bone loss associated with dental implants.

Copper also plays a vital role in bone health. It supports collagen formation [2,3], promotes the development of connective tissue, and is involved in various enzymatic reactions critical for bone metabolism [4]. A brief report by Dr. Pasco et al. analyzed the relationship between dietary copper and selenium intake and bone mineral density (BMD) in 575 women. The research reveals that insufficient copper intake is significantly associated with reduced BMD across multiple skeletal sites [5]. Despite the small impact on BMD, the findings are significant, suggesting that dietary recommendations for these trace elements may be crucial for maintaining optimal bone health in women. Additionally, another study by Dr. Han and her team explored the relationship between serum copper levels and BMD among adolescents aged 12 to 19 based on data from the 2011–2016 U.S. National Health and Nutrition Examination Survey (NHANES) [6]. The findings indicated higher serum copper levels were negatively correlated with BMD in the trunk and pelvis, with a more pronounced effect observed in males. The study also found significant interactions between age and gender, suggesting that the relationship between serum copper and BMD may vary among different demographic groups. We believe these studies highlight the complex role of copper in both adolescent and adult bone health, emphasizing the importance of maintaining a balanced copper intake.

Two studies focused on osteoporosis by Dr. Kou and his team. Firstly, the potential of Citri Reticulatae Pericarpium (CRP), a traditional Chinese medicine, in treating metabolic diseases and osteoporosis, is gradually being recognized. However, the mechanisms by which CRP addresses osteoporosis in patients with type 2 diabetes remain unclear. Dr. Kou et al. through network pharmacology and molecular modeling investigates the potential of CRP in treating type 2 diabetes-related osteoporosis (T2DOP) [7]. They identified five active compounds within CRP and 63 key targets they affect. Enrichment analysis indicates that these targets are involved in estrogen, TNF, and AGE-RAGE signaling pathways, which are associated with oxidative stress and hormonal regulation. Molecular docking and dynamic simulations confirmed strong interactions between the active components of CRP and these targets, suggesting that CRP may improve T2DOP through multiple target mechanisms, thus laying the groundwork for future research.

Secondly, Dr. Kou and his team employed Mendelian randomization analysis to explore the potential causal relationship between selenium levels and osteoporosis (OP) [8]. The study included a large sample size of 4162 participants for toenail selenium and 5477 for blood selenium, alongside data from multiple GWAS databases concerning BMD. The authors concluded that selenium levels do not have a direct causal effect on the risk of developing OP. Although this result differs from some existing experimental findings [9,10,11], we believe the authors could further investigate whether selenium may influence bone health indirectly, such as through reducing oxidative stress or modulating immune function.

In a study related to bone metabolism, Dr. Huang et al. explored the effects of low-mineral water on the role of boron in bone mineralization using a mouse model [12]. They found that boron improves bone health at low exposure levels, but these benefits decrease with higher exposure. Mice drinking purified water had higher serum boron but a reduced promotion of bone minerals than those drinking tap water, suggesting that environmental factors, such as the mineral composition of drinking water, should not be overlooked when assessing the health benefits of trace elements. We believe this finding highlights the importance of considering water quality in nutritional intervention strategies, especially in regions with significant variations in water composition.

The studies presented in this Special Issue span a wide range of topics, from trace elements such as copper and boron to plant-based bioactive compounds like CRP. They revealed multiple mechanisms that impact bone health, expanding our understanding of bone-related diseases. However, they also underscore that many underlying mechanisms remain unclear, necessitating further investigation to provide new insights and solutions. Several limitations in current research deserve attention. First, the applicability of results from animal models to humans is limited due to differences in bone metabolism and physiological conditions between species [13,14]. Therefore, future research should focus on large-scale clinical trials to validate the reliability and feasibility of these experimental findings. While existing studies have examined the individual effects of trace elements, the comprehensive impact of diet, lifestyle, and environmental factors on bone health has not been fully explored. Differences in dietary habits, activity levels, and nutritional needs among individuals highlight the need for personalized intervention strategies that account for these environmental and lifestyle variables.

Another critical area for future research is to investigate the long-term effects of trace elements and determine their optimal intake levels. Complex interactions between different elements may lead to synergistic or antagonistic effects, where excessive intake of one element could disrupt the metabolic balance of others, negatively impacting bone health. Thus, further studies are essential to explore these interactions and establish appropriate intake standards. At the same time, environmental factors, such as the mineral content of drinking water, also significantly influence the absorption and metabolism of trace elements. This finding suggests that nutritional strategies should incorporate considerations regarding water quality, particularly in regions where water composition varies. Future research should adopt a holistic, comprehensive approach that considers diet, exercise, environmental factors, and trace element intake to support the prevention and management of bone diseases.
